# Data‐Driven Molecular Typing: A New Frontier in Esophageal Cancer Management

**DOI:** 10.1002/cam4.70730

**Published:** 2025-02-28

**Authors:** Yue Du, Bianli Gu, Linlin Shi, Yong She, Qi Zhao, Shegan Gao

**Affiliations:** ^1^ Henan Key Laboratory of Microbiome and Esophageal Cancer Prevention and Treatment, Henan Key Laboratory of Cancer Epigenetics, The First Affiliated Hospital (College of Clinical Medicine) of Henan University of Science and Technology Cancer Hospital Luoyang Henan China; ^2^ State Key Laboratory of Oncology in South China, Guangdong Provincial Clinical Research Center for Cancer Sun Yat‐Sen University Cancer Center Guangzhou Guangdong China

**Keywords:** big data, esophageal squamous cell carcinoma, molecular typing, precision treatment, single‐cell sequencing

## Abstract

**Background:**

Esophageal squamous cell carcinoma (ESCC) is a predominant and highly lethal form of esophageal cancer, with a five‐year survival rate below 20%. Despite advancements, most patients are diagnosed at advanced stages, limiting effective treatment options. Multi‐omics integration, encompassing somatic genomic alterations, inherited genetic mutations, transcriptomics, proteomics, metabolomics, and single‐cell sequencing, has enabled the identification of distinct molecular subtypes of ESCC.

**Method:**

This article systematically reviewed the current status of molecular subtyping of ESCC based on big data, summarized unique subtypes with differing treatment responses and prognostic outcomes.

**Result:**

Key findings included subtype‐specific genetic mutations, signaling pathway alterations, and metabolomic profiles, which offer novel biomarkers and therapeutic targets. Furthermore, this review discusses the link between molecular subtypes and immunotherapy efficacy, chemotherapy response, and drug development.

**Conclusion:**

These insights highlight the potential of omics‐based molecular typing to transform ESCC management and facilitate personalized treatment strategies.

## 1 Introduction

Over the years, improvements in living standards and shifts in dietary habits have coincided with a gradual increase in the incidence and mortality rates of esophageal cancer. Globally, esophageal cancer ranks seventh in incidence and sixth in mortality [1], with a 5‐year survival rate of only 20% [2]. The subtle nature of esophageal cancer symptoms underscores the critical need for timely diagnosis and screening. Esophageal squamous cell carcinoma (ESCC) is the predominant histologic form of esophageal cancer, with incidence and death rates in China being higher than in other Asian and worldwide areas. Tumor heterogeneity [3], driven by genetic mutations, hereditary traits, and epigenetic alterations, complicates molecular classification, contributes to variable clinical outcomes, and hampers the identification of reliable prognostic biomarkers [4].

As biological science enters the era of genome‐wide big data, numerous innovative methods and concepts have emerged in treating contemporary tumors. Traditional clinicopathologic typing methods (e.g., histologic type and depth of infiltration) cannot meet the needs of modern precision medicine. In 1991, the National Cancer Institute (NCI) introduced the concept of molecular typing of tumors [5]. Molecular typing can reveal the heterogeneity of tumors by analyzing gene expression profiles, mutation profiles, and other biomolecular data of esophageal squamous carcinoma (ESCC) cells, which can help understand the complexity and diversity of tumors and provide patients with more precise diagnostic recommendations and treatment plans. Molecular typing of esophageal cancer provides a powerful resource for discovering potential diagnostic markers and therapeutic targets for future esophageal cancer treatment.

This study focused on molecular typing models developed using diverse technical approaches, algorithms, and cohorts. Recent research has identified molecular subtypes of esophageal cancer corresponding to various stages of disease progression. These subtypes, defined by distinct molecular characteristics, provide insights into tumorigenesis, growth, and their effects on patient survival. These findings represent significant advancements in understanding the molecular biology of esophageal cancer and establish a foundation for more precise therapeutic strategies.

## 2 Subtyping of ESCC Based on Bulk Omics Data

### 2.1 Genomics of ESCC

Despite extensive research on the esophageal cancer genome in China, most studies involve sample sizes ranging from tens to just over a hundred cases, concentrating primarily on exon sequencing of DNA coding regions. Thus, it is infeasible to attain a comprehensive picture of the genetic profile of esophageal cancer, and it is even more challenging to precisely describe how genes influence the disease progression of a patient and the confirmation of the diagnosis. However, solid tumors frequently include DNA copy number aberrations (CNA) in their genome, which are closely related to the initiation and progression of these tumors [6]. This variability spans the entire chromosome length down to regions shorter than 100 kb. The heterogeneity of tumor genomic CNA has been demonstrated through molecular cytogenetic studies [7].

Song et al. [8] carried out in‐depth exploratory research in March 2014 by integrating genomic and clinical data from Chinese patients with ESCC. They succeeded in identifying eight specific genetic variations that may contribute to the development of the disease. Six of these genetic variations have been shown to be associated with key cancer‐associated genes, including both oncogenes and tumor suppressor genes, *TP53*, *RB1*, *CDKN2A*, *PIK3CA*, *NOTCH1*, *NFE2L2*, and two additional genes not mentioned in previous reports, *ADAM29* [9] and *FAM135B*. Notably, *FAM135B* is the first identified specifically in this study to be potentially implicated in ESCC. In addition, data on copy number variations were obtained, demonstrating that MIR548K, located in the amplified region of chromosome 11q13.3–13.4 [8, 10, 11], is associated with attributes of disease progression. These mutations and copy number variations are closely related to esophageal cancer, which marks a breakthrough in the development of new drugs specific to this disease. Key protein regulatory genes implicated in lesion formation have also shown new insights in this trial. Specifically, *MLL2*, *ASH1L*, *MLL3*, *SETD1B*, and *CREBBP/EP300* showed a high frequency of non‐silent mutations [8, 12]. After further exploration of some molecules that may become new targets for future anticancer therapies, it was found that there is a very high probability of mutation in the PI3K pathway, and *PSMD2*, *RARRES1*, *SRC*, *GSK3β*, and *SGK3* are also considered potential drug targets [13, 14]. Scientists have summarized and analyzed a series of data on mutations and copy number changes of genes related to the pathogenesis of ESCC, which led to the clarification of some key signaling pathways, such as Wnt, cell cycle, Notch, RTK‐Ras, and AKT [15], which may greatly improve the therapeutic strategies for ESCC. In addition, in October 2020, Cui et al. [16] revealed some important genetic features by comparing adjacent normal tissue samples from 508 ESCC patients from Shanxi and Xinjiang with their clinical follow‐up records. Using genomic data, esophageal cancers were classified into distinct subtypes. Patients with mutations in the NFE2L2 (NRF2) pathway [17, 18] or expansions in the RTK‐RAS‐MYC pathway demonstrated poorer prognoses [19, 20]. This was particularly evident in cases with NRF2 mutations. However, patients lacking these specific mutations or pathway amplifications tended to have better survival outcomes. These findings also offer valuable insights for the development of novel therapeutic strategies. Furthermore, immunotherapy has ushered in a transformative era in cancer treatment [21]. Immune checkpoint inhibitors (ICIs) have demonstrated efficacy in advanced ESCC patients who are resistant to chemotherapy, offering a promising alternative for improved patient outcomes [22, 23, 24, 25, 26, 27]. In April 2023, Chen et al. [28] performed whole exome sequencing of tumor samples from 486 patients in the JUPITER‐06 study [29], identifying several other favorable immunogenic features (e.g., HLA‐I/II diversity [30, 31]) and risky oncogenic alterations associated with the efficacy of chemotherapy + anti‐PD‐1 (e.g., PIK3CA [32] and TET2 mutations [33]). The esophageal cancer genomic immuno‐oncology classification (EGIC) scheme was developed by integrating immunogenic features with oncogenic alterations. This classification stratifies patients into three subgroups: EGIC1 (favorable immunogenic features and absence of oncogenic alterations), EGIC2 (either favorable immunogenic features or absence of oncogenic alterations), and EGIC3 (unfavorable immunogenic features with the presence of oncogenic alterations). Clinical outcomes demonstrated that the combination of chemotherapy and anti‐PD‐1 therapy significantly improved survival in the EGIC1 and EGIC2 subgroups. However, this therapeutic approach did not yield survival benefits in the EGIC3 subgroup. Thus, EGIC may guide future individualized treatment strategies and inform mechanistic biomarker studies of chemotherapy + anti‐PD‐1 therapy in patients with advanced ESCC. Several randomized clinical trials have further demonstrated that chemotherapy + PD‐1 blockade (chemotherapy + anti‐PD‐1) combination therapy significantly improves overall survival (OS) in patients with untreated advanced ESCC [29, 34, 35, 36, 37], thus establishing chemotherapy + anti‐PD‐1 as the new standard first‐line treatment for advanced ESCC. However, not all patients respond equally to chemotherapy + anti‐PD‐1, highlighting the need to identify predictive markers to guide patient selection and discover new therapeutic targets.

Genomics studies have provided a rich data resource for the molecular typing of ESCC. By examining mutations, chromosomal alterations, and gene expression profiles, numerous key genes and signaling pathways associated with the initiation and progression of ESCC can be identified. However, genomics has its limitations. While gene sequences can be identified, there remains a lack of comprehensive understanding regarding the functions and regulatory mechanisms of many of these genes.

### 2.2 Transcriptomics of ESCC

In addition to genomic sequencing, transcriptomic‐based research in esophageal cancer tissues revealed the changes and regulatory mechanisms of esophageal cancer cells at the molecular level. This RNA‐Seq technology can help us understand the developmental process of esophageal cancer, molecular typing, prognostic assessment, and discovery of new therapeutic targets. In July 2023, Liu et al. [38] applied digital spatial analysis (DSP) to perform spatial whole transcriptome analysis on 19 patient specimens with low‐grade intraepithelial neoplasia (LGIN), high‐grade intraepithelial neoplasia (HGIN), or ESCC. Spatial transcriptome mapping of epithelial cells, immune cells, and their nuclei was performed using PanCK, CD45, and Syto13, respectively. This analysis targeted specific regions of tissue samples at different stages of ESCC progression: NE (normal), LGIN, HGIN, and ESCC. A study has demonstrated that the aberrant expression of squamous epithelial heat shock protein 58 (Cornulin, CRNN) [39] and transgelin 2 (TAGLN2) is associated with the progression of esophageal precancerous lesions to esophageal cancer. These proteins show potential as early intervention markers for esophageal cancer. Spatial whole‐transcriptome mapping has been employed to identify indicators predictive of ESCC risk at the esophageal squamous precancerous lesion (ESPL) stage and to explore the pathogenesis underlying the transition from ESPL to ESCC. The findings suggest that TAGLN2 promotes ESCC progression, while CRNN inhibits ESCC progression by regulating cell proliferation. These results provide valuable insights into the pathological processes driving ESCC development and offer a basis for early warning of ESCC and contribute to the prevention, detection, and intervention strategies for esophageal cancer. Yang et al. [40] performed single‐cell transcriptome sequencing in the same year on a cohort of esophageal squamous cancer patients with a poor response to neoadjuvant therapy and analyzed individuals receiving surgery‐only treatment for ESCC. Through these analyses, the researchers identified 13 tumor cell subpopulations distributed across different neoadjuvant therapy samples that may have resistance characteristics. The team identified a subpopulation of Ep‐C2 tumor cells with strong antioxidant characteristics in samples from patients treated with radiotherapy combined with immunotherapy. This subset showed significantly activated antioxidant‐related transcription factors MAFG and NFE2L2 and antioxidant‐related genes, including *OSGIN1* and *CYP4F3*. This suggests that the antioxidant response may have a potential role in tolerance to radiotherapy combined with immunotherapy. Subsequently, multiple immunohistochemistry experiments confirmed that the activation of antioxidant‐related markers and proteins was also present in an independent cohort of patients receiving radiotherapy combined with immunotherapy. Based on the genetic characterization of Ep‐C2, the team further explored potential drugs that might inhibit Ep‐C2 cell activity to improve the efficacy of radiotherapy combined with immunotherapy. In addition, they analyzed the detailed characteristics of T cells, myeloid cells, endothelial cells, and fibroblasts under different neoadjuvant regimens and found that the activation and effector functions of T cells were suppressed during neoadjuvant therapy and proposed the possibility of using IL15 agonists as a combination therapy to boost immunological effects.

The developmental mechanisms of esophageal cancer can be thoroughly understood by transcriptomics. A thorough analysis of the variations in gene expression between cancerous and normal tissues can reveal the key molecular pathways of disease progression and provide potential targets and protocols for precision therapy. Transcriptomics, however, primarily focuses on the transcriptional level, with a limited understanding of processes such as post‐transcriptional modification, protein translation, and degradation. The tumor microenvironment (TME) of esophageal cancer is very complex, and a single transcriptomic analysis may not fully reveal the interactions between the tumor and the host.

### 2.3 Proteomics and Metabolomics of ESCC

Genomic alterations often manifest as protein expression changes, further regulated by post‐translational modifications (PTMs). Thus, proteomics and phosphorylated proteomics analyses may provide additional insights into tumor biology that genomic analyses cannot decipher, and proteomics is emerging as a hot topic in esophageal cancer research.

In August 2021, Liu et al. [41] conducted a comprehensive study involving 124 pairs of ESCC tumor tissues and adjacent non‐tumor tissues, analyzed using TMT‐tagged quantitative proteomics. Moreover, phosphorylated proteomics without tagging was applied to 31 of these sample pairs, revealing dysregulated proteins and pathways in ESCC tumor tissues. Two molecular subtypes were identified through consensus clustering of the proteomic data, which may have prognostic significance for patient survival outcomes. These subtypes were classified as the lower‐risk S1 and higher‐risk S2 groups based on large‐scale proteomic and phosphoproteomic analyses of the molecular characteristics of esophageal cancer. Based on ELOA [42] and SCAF4 [43, 44], a model for confirming the diagnosis and evaluating progression was developed. Next, 295 immunohistochemical samples from tumor tissues were applied to the model for validation. Three potentially effective drugs for patients with type S2, including menadione, GW8510, and sulconazole, were also predicted, and a series of biological function tests confirmed their significant inhibitory effects on esophageal cancer cells. In November 2021, Li et al. [45] selected 94 surgically resected primary tumor tissue samples (T) and 24 non‐tumor esophageal tissue control samples (N) from 94 patients with moderately advanced (TNM stage II‐IV) ESCC and performed extensive proteomic and phosphoproteomic characterization using iTRAQ technology, which identified a total of 9042 proteins as well as 26,892 phospho‐sites. A total of 556 differentially expressed proteins (DEPs) were found, of which 227 were upregulated and 329 downregulated. Moreover, 1691 differentially expressed phosphorylation sites (DEPSs) were identified, of which 695 were upregulated and 996 downregulated. The study defined three key protein group isoforms in the ESCC tumor cohort: S‐I, S‐II, and S‐III. The S‐III isoform was shown to have the highest proportion of patients with lymph node metastases and the significantly lowest disease‐free survival (DFS). Furthermore, in this phenotype, there were higher levels of proteins and phosphorylated proteins in the spliceosome pathway. The PP1 inhibitors CD2BP2 and WBP11 [46, 47], acting as CLK1 kinase enhancers, were upregulated in S‐III fractional ESCC and functioned in spliceosome assembly and pre‐mRNA splicing, and knockdown of CD2BP2 and WBP11 significantly inhibited the proliferation of ESCC cells. These findings provide important evidence for CLK1 as a potential and therapeutically promising target, which may help us to develop new therapeutic regimens to improve the prognosis of ESCC patients.

Proteomic analysis, proteomic subtype definition, diagnostic and prognostic model construction, drug prediction, and validation analyses were used to define the subtypes of ESCC and their characteristics, highlighting the important value of proteomic data for disease research and providing a molecular basis for finding potential therapeutic approaches for ESCC. The presence of multiple PTMs and splice variants complicates the identification and quantification of proteins.

### 2.4 Metabolomics of ESCC

Metabolic reprogramming is a significant hallmark of cancer and plays a role in both the development and progression of cancer [48]. Metabolomics studies of esophageal cancer analyzed metabolite changes in tumor tissues and body fluids, providing insights into disease mechanisms and new avenues for early diagnosis and treatment. Assessment of cancer metabolism will help improve understanding of cancer biology and support the identification of cancer biomarkers and the discovery of new cancer targets [49, 50]. Significant changes in the amounts of metabolites, including amino acids, lipids, glucose, and nucleotides, have been shown in ESCC by several tissue‐based metabolomics investigations [51, 52, 53, 54]. These results suggest that metabolic pathways may provide the basis for biomarker discovery in ESCC [55, 56, 57, 58]. In January 2021, Chen et al. [59] performed the metabolomic analysis of 141 ESCC cancer tissue samples and 70 non‐cancer tissue samples by ultra‐performance liquid chromatography combined with high‐resolution mass spectrometry (UPLC/MS). A total of 41 differential metabolites were identified, and 37 were validated in the test dataset. Kaplan–Meier survival analysis and Cox proportional risk regression analysis demonstrated that specific metabolites (e.g., 2‐hydroxymyristoylcarnitine, 3‐hydroxyhexadecanoylcarnitine, and 2,3‐Dinor‐TXB1) were significantly associated with overall survival (OS). Amino acid transporters, such as SLC7A5/LAT1 [60], SLC1A5/ASCT2 [61], and SLC16A10/MCT10 [62], were upregulated in ESCC tissues. These amino acid transporters were expressed at higher levels in malignant tissues than in non‐cancerous tissues, according to immunohistochemical (IHC) staining. This study not only identified several metabolites of diagnostic and prognostic value but also provided an accurate metabolite‐based predictive model for ESCC tissue classification and identified three upregulated amino acid transporters [63, 64, 65, 66] as potential therapeutic targets for ESCC, especially SLC1A5. Lv et al. [67] concentrated on the metabolomic characterization of ESCC and the establishment of early screening models. In partnership with the Chinese Academy of Sciences' Shanghai Laboratory of Organic Chemistry and the Shandong Cancer Prevention Center, an experimental platform covering the whole disease process tracking has been successfully built—“National Demonstration Base for Early Diagnosis and Treatment of Esophageal Cancer (Feicheng City, Shandong Province).” An article published in the May 2021 issue of *Clinical and Translational Medicine* detailed the analysis of a dataset comprising 1104 samples with complete pathology and metabolomic profiles. The study identified metabolic markers that hold potential for esophageal cancer screening and the early detection of high‐risk groups. This strategy offers a promising approach for esophageal cancer screening in China's high‐incidence regions. Its effectiveness has been evaluated using an independent validation set and actual esophageal cancer cases. The model demonstrated a high area under the curve (AUC) of 0.81 in the validation set, surpassing the AUC of 0.64 achieved by traditional risk factor assessment models, with a positive predictive value reaching 20%. These findings highlight the high accuracy and predictive value of metabolomic markers in early esophageal cancer screening. Furthermore, the research team analyzed 653 samples with comprehensive baseline data and metabolomic profiles, identifying 15 metabolites, including histidine and tryptophan, associated with ESCC progression and showing a monotonic trend with disease progression.

Several metabolites are structurally complex, and there are difficulties in identification and quantification. The changing trends of these metabolites provide new perspectives for understanding esophageal cancer's biological properties and pathological mechanisms. The quality of life and survival rate of patients with esophageal cancer are anticipated to be considerably enhanced by in‐depth metabolomics studies.

### 2.5 Multi‐Omics Studies of ESCC

In the study of esophageal cancer, multi‐omics methods, including transcriptomics, proteomics, and phosphoproteomics, have been extensively used. Multi‐omics analysis has uncovered the molecular heterogeneity of esophageal cancer, developed a molecular classification system for ESCC, and facilitated the discovery of novel therapeutic targets.

In December 2022, Liu et al. [68] conducted comprehensive whole‐genome sequencing (WGS), whole‐genome bisulfite sequencing (WGBS), RNA sequencing (RNA‐Seq), small RNA sequencing (sRNA‐Seq), and proteomics analysis on samples from 155 patients with ESCC and adjacent normal tissues. Through a cluster of cluster assignments (COCA) analysis [69], ESCC was classified into four distinct molecular subtypes: cell cycle signaling activation (CCA) subtype, NRF2 oncogenic activation (NRFA) subtype, immunosuppression (IS) subtype, and immunomodulation (IM) subtype. Scientists identified each ESCC molecular subtype's traits and potential treatment targets during the analytical process. For example, 84.6% of the CCA subtype samples had mutations at the gene level directly related to cell cycle regulation [8], including 11q13.3/CCND1 amplification and CDKN2A/B homozygous deletion. Follow‐up studies revealed that patients with genetic variants associated with cell cycle control responded more strongly to palbociclib (a drug targeting cyclin D kinase 4 and 6 inhibitors). Using multi‐omics techniques to dig deeper into the key factors, they found that in addition to changes, such as overexpression or deletion of NFE2L2, a key protein of the NRF2 pathway, hypermethylation of KEAP1 and amplification of SOX2 [70], both of which also lead to activation of the pathway, are also important mechanisms. The other two important types are IS and IM, which show high levels of immune cell infiltration, suggesting that treatment could be with immunotherapy. The IS subtype is characterized by highly active B cells and NK CD56 bright cells, with its regulatory region located in the “CD4 + T cm − Tem.” This suggests that combining immunotherapy with targeted HER2 therapy enhances the probability of a favorable outcome. Another important observation was the infiltration of abundant macrophages and CD8^+^ T cells in the IM subtype, with the primary immune microenvironment module being “CD8^+^ T‐Macrophage.” Most patients who responded well to PD1 treatment belonged to the IM subtype, with only a few cases demonstrating deterioration after PD1 therapy. The majority of patients who responded effectively to PD1 treatment were of the IM subtype, with only a small number experiencing deterioration following therapy. This outcome provides a fresh perspective for devising tailored treatment strategies for this patient group and catalyzes further drug development. In April 2024, Zhao et al. [71] sequenced the tumor tissues of 60 patients with preoperative untreated ESCC by transcriptomics, proteomics, and phosphoproteomics. Based on different molecular features, ESCC can be classified into three subtypes with different clinical characteristics, among which type S‐III has the worst prognosis and in which pathways, such as glycolytic pathway and DNA repair are significantly enriched. Furthermore, TIMMDC1, an assembly factor for mitochondrial complex 1, may be a potential prognostic molecule for ESCC. Kinase‐substrate network analysis obtained based on phosphorylated proteomic data predicted candidate kinases, in vitro and in vivo experiments further confirmed that casein kinase II subunit alpha (CSNK2A1) could serve as a potential kinase target in ESCC. This study offers novel molecular typing and therapeutic targets for ESCC, advancing understanding of the biological characteristics and intervention targets of this disease.

The analysis highlights that single‐cell studies, combined with genomics, transcriptomics, proteomics, metabolomics, and multi‐omics approaches, have significantly advanced the understanding of the molecular mechanisms underlying esophageal cancer. These methodologies have identified key biomarkers and therapeutic targets, contributing to the development of precision medicine. Such advancements facilitate the formulation of more effective treatment strategies, enhancing therapeutic outcomes and improving patient prognoses (Table 1). Despite these achievements, technical and analytical challenges remain. Continued research and technological innovation are expected to progressively overcome these limitations, paving the way for personalized treatment plans tailored to specific molecular subtypes. This progress has the potential for the realization of precision treatment in esophageal cancer.

**TABLE 1:** Studies on the molecular classification of esophageal squamous cell carcinoma.

Technology	Calculation method	Sample size	Results	Clinical significance	Refs
WGS WES a‐CGH	Copy number alteration (CNA) mutation	158 ESCC patients	FAM135B is a novel oncogene. MIR548K promotes ESCC, and some key signaling pathways, such as Wnt, cell cycle, Notch, RTK‐Ras, and AKT, were identified	Some key signaling pathways were identified by relevant gene mutations and copy number changes	[8]
WGS	NMF mutation BIC‐Seq2 SCNA SMG	508 ESCC patients	Mutations in the NFE2L2 gene that were significantly associated with poor prognosis were identified, and three ESCC subtypes (NFE2L2‐mutated, RTK‐RAS‐MYC‐amplified, and double‐negative) were proposed	Clinically relevant coding and noncoding genomic alterations were revealed, and three major subtypes were identified that robustly predicted patient outcomes	[16]
WES	CNA‐corrected TMB (ccTMB)	486 ESCC patients	Found a favorable immunogenicity characteristics (HLA—I/II diversity) and tumor risk change (PIK3CA and TET2 mutation) and the effect of chemotherapy unite against PD‐1. The patients were classified into three subtypes: EGIC1, EGIC2 and EGIC3	The EGIC classification scheme can help guide future individualized treatment strategies and provide information for mechanistic biomarker studies of chemotherapy combined with anti‐PD‐1 therapy in patients with ESCC	[28]
Spatial transcriptomics	Spatial whole‐transcriptome atlas (WTA)	6 NE, 12 LGIN, 12 HGIN and 7 ESCC	TAGLN2 promotes the progression of ESCC, while CRNN regulates cell proliferation to inhibit the progression of ESCC	These indicators can help to distinguish patients with different pathological stages and provide a basis for individualized treatment of ESCC	[38]
Phosphoproteomics	PCA Hierarchical clustering Consensus clustering based on protein expression	124 paired ESCC tumor and the corresponding adjacent non‐tumor tissues	The patients were classified into two molecular subtypes: S1 and S2	Potential treatment strategies were provided for S2 subtype patients through subtype‐specific drug prediction and validation. Drugs, such as GW8510, menadione, and sulconazole, were validated to inhibit the growth of ESCC	[41]
Phosphoproteomics	PCA Consensus clustering DEPs DEPSs DEPPs PIPs PRA	94 surgically resected primary tumor tissues (T) and 24 non‐tumor esophageal tissues (N)	Three major proteomic subtypes (S‐I, S‐II and S‐III) in this tumor cohort of ESCC were defined	CLK1 is a promising new ESCC treatment target, especially in poor prognosis after surgery S‐III subtypes	[45]
Metabolomics	PCA PLS‐DA SVM RF	141 ESCC cancerous tissue samples and 70 non‐cancerous counterparts	The amino acid transporters SLC7A5/LAT1, SLC1A5/ASCT2 and SLC16A10/MCT10 were upregulated in ESCC cancerous tissues	The three upregulated amino acid transporters were identified as potential therapeutic targets for ESCC, especially SLC1A5	[59]
Metabolomics	PCA PLS‐DA RF MetaboAnalyst	1104 participants	Metabolites related to the occurrence and development of ESCC, such as histidine, were screened	Metabolic biomarkers of esophageal squamous cell carcinoma were screened, and the relevant metabolic pathways related to the occurrence and development of esophageal squamous cell carcinoma were studied, providing important evidence for early diagnosis and treatment of esophageal cancer	[67]
WGS WGBS RNA‐Seq sRNA‐Seq Proteomics	Consensus clustering RF SMOTE	155 ESCC patients	ESCCs were classified into four molecular subtypes: CCA, NRFA, IS, and IM	Multiple omics analysis of ESCC provides a comprehensive molecular classification, which provides potential therapeutic targets for each subtype or diagnostic biomarker, helping to improve the understanding and treatment of ESCC	[68]
Transcriptomic Proteomic Phosphoproteomic	Unsupervised clustering analysis of the proteomics data silico kinome activity profiling (iKAP)	60 paired treatment‐naive ESCC and adjacent non‐tumor tissue samples	ESCC were classified into three subtypes with different clinical features: S‐I, S‐II, and S‐III	Translocase of the inner mitochondrial membrane domain containing 1 (TIMMDC1) was validated as a potential prognostic molecule for ESCC, and casein kinase II subunit alpha (CSNK2A1) as a potential kinase target for ESCC	[71]
scRNA‐Seq	NMF	208,659 single‐cell transcriptomes derived from 60 ESCC patients	Findings that TME in ESCC is enriched in Treg and TEX but deficient in TEFF and TMEM; revelation of the activation of CAFs in the development of ESCC; significant correlation between the level of gene expression in the mucosal immune‐like program and the survival time of patients with ESCC and the effect of somatic mutations (e.g., TP53 and NOTCH1)	This study provides a detailed map of the ESCC ecosystem, revealing complex interactions between cancer cells, stromal cells, and immune cells, which provides important information for an in‐depth understanding of the pathogenesis and progression of ESCC and may contribute to the development of precision medicine approaches	[72]

## 3 Single‐Cell Studies of ESCC

In recent years, single‐cell technology has continuously evolved, providing unprecedented opportunities to decipher the biological characteristics of individual cells. This technology effectively addresses the challenge of cellular heterogeneity and elevates the precision and accuracy of research to new heights with its robust technical advantages, thereby facilitating the precise identification of molecular subtypes of ESCC.

In September 2021, Zhang et al. [72] collaborated to study the composition of ESCC tumors using 208,659 single‐cell transcriptomes from 60 individuals. Their analysis identified eight common expression programs in malignant epithelial cells and 42 distinct cell types, comprising 26 immune cell subtypes and 16 non‐immune stromal cell subtypes within the TME. Interactions between cancer cells and other cellular populations and interactions among various cell types within the TME were systematically analyzed [73, 74, 75]. In addition, the study associated the cancer cell transcriptome with somatic mutations and identified several markers significantly associated with patient survival, which may be relevant to the precise treatment of ESCC patients. A comprehensive picture of the ESCC tumor ecosystem was provided by single‐cell transcriptome analysis, offering new perspectives and potential targets for future research and therapy. In March 2023, Chen et al. [76] investigated the activation of fibroblasts by epithelial cells to promote the development of esophageal cancer. The study performed single‐cell RNA sequencing (scRNA‐Seq) and spatial transcriptome analysis on 79 samples from 29 ESCC patients, including samples of tumors and their adjacent normal tissues (NOR), LGIN and HGIN [77]. The study revealed compositional and transcriptional changes in various cell types (e.g., epithelial cells, fibroblasts, endothelial cells, T‐cells, B‐cells, myeloid cells, and mast cells) in different disease stages. ANXA1 expression in epithelial cells was found to be progressively and significantly lost during lesion progression due to inhibition of the transcription factor KLF4 [78]. ANXA1 [79] deficiency resulted in the uncontrolled transformation of normal fibroblasts into cancer‐associated fibroblasts (CAFs), which could be augmented by TGF‐β secreted [80, 81] by malignant epithelial cells. With ESCC progression, the expression level of ANXA1 in epithelial cells gradually reduced, revealing a novel mechanism of CAF activation in the ESCC TME. In October 2023, Liu et al. [82] collaborated in their single‐cell RNA sequencing of tumors from ESCC patients undergoing neoadjuvant immune checkpoint blockade (ICB), which revealed a subpopulation of depleted CD8^+^ T cells expressing SPRY1, which exhibited a progenitor‐depleted T‐cell (Tpex) phenotype and were associated with a response to ICB complete response [83]. Progenitor‐like depleted SPRY1^+^CD8^+^ T cells enhanced ESCC responsiveness to neoadjuvant PD‐1 blockade. The findings of the research provided a new direction for the future of tailored immunotherapy by systematically analyzing the cellular and molecular characteristics of the population with differential immunotherapy efficacy in ESCC, revealing the key molecular mechanism of depleted precursor T cells in ESCC as a response to immunotherapy and proposing it as a biomarker for predicting the efficacy of immunotherapy.

Single‐cell studies deepen understanding of the complex mechanisms underlying tumor formation and drive the advancement of tailored medicine and precision treatment strategies, facilitating the design of more effective therapeutic regimens for various subtypes of ESCC. However, they are constrained by challenges in integrating diverse biological data and translating findings into clinical applications.

## 4 Combined Single‐Cell Multi‐Omics Studies of ESCC

Single‐cell studies can reveal the gene expression and functional status of individual cells, describe in detail the TME [84, 85], including the interactions of immune cells and stromal cells, and discover rare cellular subpopulations and functional states, and have unique advantages in gaining insight into cellular information [86]. Genome, transcriptome, proteome, and metabolome are commonly studied molecular profiles [87]. Researchers have attempted to integrate multi‐omics data to achieve a more comprehensive understanding of the pathogenesis of esophageal cancer. Multi‐omics encompasses a diverse collection of heterogeneous data, highlighting temporal changes in various biomolecules, including proteins, metabolites, and genes, across multiple biological levels. According to the research results, there are about 20,000–22,000 protein‐coding genes [88], 20,000 proteins [89], and more than 114,100 metabolites [90] in the human body. This vast and complex biological repertoire exemplifies the defining characteristics of “big” data, which cannot be overlooked. To better address research demands, multi‐omics databases such as the Cancer Genome Atlas (TCGA) and the International Cancer Genome Consortium (ICGC) are continuously expanding, integrating additional components and samples to improve their utility and comprehensiveness.

Multi‐omics data demonstrate the complexity of many cancer developments, and building such models across multi‐omics data has become an urgent problem. The combined use of single‐cell and multi‐omics data can provide in‐depth information for the systematic understanding of tumor biological processes, and with the all‐encompassing information it provides, it is possible to improve the accuracy of tumor prognosis, thus facilitating more effective tumor diagnosis and treatment [91]. Therefore, researchers at home and abroad have begun to use single‐cell and multi‐omics data to carry out work on molecular typing of cancer, determination of markers, and so on, and have developed a series of methods. However, the current direct use of multi‐omics data for molecular typing of esophageal cancer is still relatively understudied, which prevents us from deeply understanding the molecular typing principle of ESCC.

## 5 Clinical Relevance of Molecular Typing in the Chinese Population

The molecular typing of esophageal cancer in the Chinese population is significantly different from that of the Western population, which is mainly manifested in gene mutations, gene expression profiles, and copy number variations. These differences reflect how populations differ regarding their genetic background, way of life, and environmental exposure.

Genomic studies have significantly advanced the understanding of molecular mechanisms in ESCC by analyzing genetic variations and molecular signatures. Complementary omics approaches, including transcriptomics, proteomics, and metabolomics, provide key insights into the biology of ESCC. Transcriptomics focuses on gene expression levels and regulatory mechanisms, proteomics investigates protein expression and function, and metabolomics uncovers biochemical changes associated with tumor progression. Moreover, single‐cell analysis allows the identification of distinct tumor cell subsets and states, including rare cell populations, increasing the understanding of their roles in tumor development, metastasis, and treatment responses. These integrated studies offer a comprehensive view of ESCC's molecular landscape and facilitate the discovery of disease‐related biomarkers and therapeutic targets.

In terms of clinical application, molecular typing based on big data can provide more precise treatment options for patients with ESCC, especially in a country with a high incidence of esophageal cancer, such as China. Evaluating the molecular typing of patients allows for more precise predictions of their responses to various treatments, enabling the selection of the most appropriate treatment plan. This approach also reduces the likelihood of side effects and drug resistance, further enhancing treatment efficacy.

## 6 Computational Improvement for the Subtyping

Data preprocessing is a critical step in esophageal cancer molecular typing studies to reduce noise and errors in the data. This includes processing missing values, normalizing data, and providing high‐quality input data for subsequent analysis. Molecular feature screening is usually based on the coefficient of variation (CV), absolute median difference (MAD), standard deviation (SD), and other computational indicators to select features with significant expression kurtosis fluctuations between different samples to improve the computational efficiency of downstream typing algorithms. As molecular typing is unsupervised, the algorithm needs to be run multiple times to assess the clustering stability of different class numbers. This typically involves generating a clustering consistency matrix and utilizing tools, such as heat maps, consensus cumulative distribution functions, and so on, to help determine the optimal number of subtypes. The ConsensusCluster algorithm facilitates consistent clustering by allowing users to specify parameters, including the maximum number of clusters and the number of resamples. The non‐negative matrix factorization (NMF) algorithm is employed to extract key features and predict the isotype of samples. Visualization tools, such as heatmaps, are used to display molecular typing results, providing insights into the relationships between different subtypes and their associations with clinical features. Following molecular typing, clinical characteristics, pathological stages, and survival statuses of various subtypes can be analyzed. Moreover, differential gene expression analysis and survival analysis can be conducted to further explore the biological and clinical implications of the identified subtypes.

With the development of high‐throughput sequencing technology, a large amount of omics data related to esophageal cancer has been accumulated. Researchers have developed new computational models to analyze molecular data from esophageal cancer, including machine learning and bioinformatics tools that can reveal molecular subtypes of esophageal cancer and assess prognosis. Computational methods for molecular typing are constantly improving, providing powerful tools and methods for achieving precision treatment.

## 7 Challenges and Future Directions

### 7.1 Challenges

Firstly, the volume and complexity of the data present a significant challenge. Multi‐omics technologies, such as genomics, transcriptomics, proteomics, and metabolomics, generate vast amounts of biological data, necessitating robust computational power and advanced bioinformatics tools for storage, processing, and analysis. This demands highly developed technical capabilities and extensive experience in data processing. Secondly, integrating data from various histological levels poses a challenge. These data possess distinct characteristics and scales, and effectively integrating these datasets to extract valuable insights remains a key technical hurdle. Moreover, ensuring data standardization and comparability across different studies is an ongoing concern. Finally, translating research findings into clinical practice presents another challenge. The ultimate goal of multi‐omics research is to guide clinical decisions and improve treatment strategies. How to effectively bridge the gap between basic research and clinical application, particularly in molecular typing and personalized treatment, remains a pivotal area of focus.

### 7.2 Future Directions

Future research should prioritize the integration of multi‐omics techniques to achieve a more comprehensive understanding of the molecular mechanisms underlying esophageal cancer. Interdisciplinary approaches will play a pivotal role in advancing efforts in molecular typing. Innovative approaches to data analysis are essential, and advancements in machine learning and artificial intelligence technologies promise to make future methods more intelligent and automated. Leveraging these technologies is anticipated to improve the efficiency and accuracy of data analysis while enabling the extraction of valuable insights from complex datasets. Future studies should prioritize clinical samples and prospective clinical trials to establish the clinical relevance of molecular typing. A deeper understanding of the molecular mechanisms driving esophageal cancer will pave the way for the development of targeted therapeutic agents and treatment regimens tailored to specific molecular subtypes, ultimately achieving precision medicine. In addition, research into the molecular staging of esophageal cancer must involve global collaboration and data sharing. International cooperation will be crucial for integrating diverse samples and data, thereby enhancing the statistical power of studies and the broader applicability of results.

In summary, while multi‐omics research holds immense promise for advancing molecular typing in esophageal cancer, several challenges remain. Future efforts should concentrate on technological innovation, clinical translation, and international collaboration to provide more precise and effective treatment options for esophageal cancer patients.

## 8 Summary and Prospects

Future advancements in gene sequencing, molecular typing, multimodal imaging, and the comprehensive analysis of clinical data through artificial intelligence will enable more precise evaluations and the development of tailored treatment plans and prognoses for esophageal cancer patients. With the continuous deepening of research and technological advances, it is reasonable to believe that molecular typing will play a crucial role in the diagnosis and treatment of ESCC. These research results promote the development of esophageal cancer treatment toward precision and individualization. For patients with advanced esophageal cancer, it is crucial to develop personalized treatment strategies based on their specific symptomatic presentation, histological type, and genomic characteristics. The ideal molecular typing approach in the future should be able to effectively deal with the heterogeneity of cancer cells, improve the accuracy of predicting disease progression, and optimize treatment planning. Therefore, we anticipate that the treatment of esophageal cancer will gradually evolve into individually tailored therapies based on molecular typing.

In the molecular typing study of esophageal cancer, the achievement of multi‐omics is outstanding. Advancements in multi‐omics have significantly contributed to molecular typing studies of esophageal cancer, offering novel perspectives and innovative methodologies for basic research while instilling renewed hope for clinical treatment. With ongoing research and technological advancements, future treatments for esophageal cancer are expected to be more precise and effective, leading to improved survival rates and quality of life for patients. Big data‐driven studies on the molecular typing of ESCC have introduced a transformative approach to precise treatment and the prediction of disease progression. Nonetheless, significant advancements are still required to achieve fully “customized” treatment plans tailored to individual patient needs.

## Author Contributions

**Yue Du:** conceptualization, methodology, formal analysis, writing – original draft, writing – review and editing. **Bianli Gu:** formal analysis, funding acquisition, writing – review and editing. **Linlin Shi:** methodology, writing – review and editing. **Yong She:** methodology, writing – review and editing. **Qi Zhao:** conceptualization, funding acquisition, project administration, writing – original draft, writing – review and editing. **Shegan Gao:** conceptualization, funding acquisition, project administration, writing – review and editing.

## Conflicts of Interest

The authors declare no conflicts of interest.

## Data Availability

Data sharing is not applicable to this article as no new data were created or analyzed in this study.
